# Redefining Soft Tissue Sarcoma Imaging: The Expanding Role of Positron Emission Tomography/Computed Tomography: A Review

**DOI:** 10.1055/s-0046-1825754

**Published:** 2026-07-15

**Authors:** Ammad S. Al Tamimi, Hamad S. Al Busaidi, Mohammed A. Al Mamari, Saif Z. Al Salmi, Hussain I. Al Bulushi, Mohammed K. Al Balushi, Salim M. Al Abri, Saleh S. Al Abri, Qsee H. Al Omairi

**Affiliations:** 1 Department of Radiology, Division of Nuclear Medicine, The Medical City for Military and Security Services (MCMSS), Al Sawadi, Sultanate of Oman; 2 Department of Medicine, Division of Medical Services, Royal Court Affairs (RCA), Al Sawadi, Sultanate of Oman

**Keywords:** soft tissue sarcoma, PET/CT, FDG, quantitative PET, radiotracers, tumor heterogeneity, radiomics, artificial intelligence

## Abstract

**Background:**

Soft tissue sarcomas (STSs) are a heterogeneous group of mesenchymal malignancies with diverse biological behavior, posing significant challenges in tumor grading, prognostic stratification, and early treatment response assessment. While magnetic resonance imaging (MRI) remains the standard modality for local tumor evaluation, its ability to characterize tumor biology and predict clinical outcomes is limited. Advanced imaging, particularly positron emission tomography/computed tomography (PET/CT), plays a critical role in improving disease characterization and supporting personalized patient management.

**Methods:**

This narrative review summarizes the role of PET/CT in STS, focusing on established and emerging radiotracers, quantitative PET metrics, and multiparametric imaging approaches, along with their clinical applications.

**Results:**

^18^ F-fluorodeoxyglucose (FDG) PET/CT remains the cornerstone of functional imaging in STS, with uptake correlating with tumor grade, aggressiveness, and prognosis. Quantitative parameters such as standardized uptake values, metabolic tumor volume, and total lesion glycolysis provide additional prognostic and predictive information. Non-FDG tracers targeting proliferation, amino acid transport, perfusion, and hypoxia offer complementary insights into tumor biology. Multiparametric PET imaging improves assessment of intratumoral heterogeneity and supports biopsy guidance, radiotherapy planning, and early treatment response evaluation. Emerging approaches, including PET/MRI, radiomics, and artificial intelligence, show promise for improving tumor characterization and predictive modeling; however, these techniques remain largely investigational and require further validation before routine clinical implementation.

**Conclusion:**

PET/CT is a key modality in STS, enabling comprehensive assessment of tumor biology and supporting personalized management. Integration of quantitative metrics, novel radiotracers, and advanced analytical techniques is expected to improve diagnostic accuracy, prognostication, and treatment planning. Further large-scale studies are required to standardize methodologies and confirm clinical impact.

## Introduction

Sarcomas are rare malignant tumors originating from mesenchymal cells and are broadly classified into soft tissue and bone sarcomas. According to the World Health Organization Classification of Soft Tissue and Bone Tumors (fifth edition), soft tissue sarcomas (STSs) comprise more than 80 histological subtypes characterized by distinct cellular origins, molecular alterations, biological behaviors, and clinical outcomes. This marked heterogeneity presents significant challenges in diagnosis, prognostication, treatment selection, and response assessment. They account for approximately 1% of adult malignancies and up to 15% of pediatric cancers. 1 2 STSs are the most common subtype and are associated with significant morbidity and mortality. Prognosis is strongly influenced by tumor grade. 2 These tumors primarily spread hematogenously, with metastatic risk determined by tumor size, grade, location, and histological subtype. 3 The lungs are the most frequent metastatic site (∼75% of cases), except for myxoid or round cell liposarcomas, which preferentially metastasize to the retroperitoneum and bone. 4

## Imaging of Soft Tissue Sarcoma

Evaluation of STS employs conventional radiography, ultrasonography, scintigraphy, computed tomography (CT), magnetic resonance imaging (MRI), and positron emission tomography/CT (PET/CT). MRI remains the gold standard for local tumor characterization, surgical planning, and assessment of tumor extent because of its superior soft-tissue contrast, multiplanar imaging capabilities, and excellent delineation of tumor relationships with adjacent muscles, fascia, bones, and neurovascular structures, making it the preferred modality for local staging of STSs. 5 6 7 PET/CT provides complementary metabolic and functional information, increasingly recognized as a valuable tool in disease assessment. 8 9 10

Given the substantial biological heterogeneity of STS and the limitations of conventional anatomical imaging in assessing tumor aggressiveness, treatment response, and prognosis, there is increasing interest in functional imaging techniques. PET/CT provides quantitative metabolic information that complements CT and MRI, supporting tumor characterization, staging, treatment monitoring, and risk stratification. Consequently, PET/CT has emerged as an important tool in the multidisciplinary management of STS. 8 9 10

PET/CT plays an important role in radiotherapy response assessment by detecting early metabolic changes that precede measurable reductions in tumor size, allowing earlier identification of responders compared with anatomical imaging criteria. 11 12 However, appropriate timing after radiotherapy is essential, as posttreatment inflammatory changes may result in transient increased FDG uptake and potentially confound interpretation. 13 14 PET/CT is also valuable in differentiating viable tumor from posttreatment changes such as edema and fibrosis, thereby improving response assessment accuracy. Furthermore, assessment of tumor viability and necrosis using metabolic imaging provides important prognostic information that is not reliably captured by conventional CT or MRI. 15 16

## Role of PET/CT in Soft Tissue Sarcoma

PET/CT, particularly with ^18^ F-fluorodeoxyglucose (FDG), has become an indispensable tool in the evaluation and management of STSs, providing unique insights into tumor metabolism and biology that complement conventional imaging modalities. By capturing glucose uptake, FDG PET/CT enables noninvasive assessment of tumor grade and aggressiveness, which is critical given the heterogeneous histology and molecular diversity of STS. 3 4 17 High FDG uptake correlates strongly with high-grade tumors and poor prognosis, as demonstrated by Schwarzbach et al and further validated in meta-analytic data. 3 4 Dynamic PET studies have shown that kinetic parameters may enhance discrimination between benign and malignant lesions, highlighting the value of metabolic assessment over morphology alone. 18

PET/CT is also instrumental in biopsy guidance, particularly in heterogeneous tumors. By identifying the most metabolically active regions, PET-directed biopsies improve diagnostic yield and reduce under-grading risk, as reported in studies. 19 20

In staging and detection of metastatic disease, PET/CT provides high sensitivity for identifying distant metastases and occult lesions. Studies showed that FDG PET/CT can reveal metastatic disease missed by CT or MRI, leading to upstaging and modification of therapeutic planning. 21 22 The lungs, bones, and soft tissues are common metastatic sites, with PET/CT outperforming bone scintigraphy in skeletal involvement. 23

PET/CT also enables early treatment response assessment, detecting metabolic changes before morphologic alterations occur. Studies demonstrated that decreases in FDG uptake after initial chemotherapy cycles correlate more accurately with histopathologic response than changes in tumor size. 20 24 This early predictive capability facilitates adaptive treatment strategies, allowing for timely modification of therapy in nonresponders. Multi-agent and multiparametric PET approaches further refine response evaluation. 25

The modality also has robust prognostic value. The baseline maximum standardized uptake value (SUVmax), metabolic tumor volume (MTV), and total lesion glycolysis (TLG) have been consistently linked to survival outcomes. 4 26 27

In restaging and surveillance, PET/CT accurately identifies local recurrence and distinguishes viable tumor from posttreatment changes, although interpretation can be limited by nonspecific uptake in inflammation or fibrosis. 28 Emerging hybrid PET/MRI improves local tumor characterization, particularly in pediatric and extremity STS, 29 30 while radiomics and artificial intelligence (AI) approaches enable quantitative feature extraction for predicting grade, response, and survival, enhancing precision medicine applications. 31

Despite limitations including nonspecific FDG uptake, partial volume effects, and variability in quantitative metrics, PET/CT remains a central imaging tool in STS. 32 33 34 Its integration with novel tracers, multiparametric imaging, and AI-driven analysis promises further refinement in personalized sarcoma care.

## PET Radiotracers in Soft Tissue Sarcoma

Several PET/CT radiotracers have been used for evaluating STSs, targeting tumor perfusion, metabolism, proliferation, amino acid transport, and hypoxia. These tracers provide complementary information to conventional imaging, aiding in tumor detection, grading, treatment monitoring, and prognostication. 1

### ^15^ O-Water

^15^ O-water measures tissue blood flow via simple diffusion. It is widely used for myocardial and cerebral perfusion and has been applied to assess skeletal muscle blood flow 35 and monitor liver metastases and the pharmacokinetics of intra-arterial and intravenous ^18^ F-fluorouracil. 36 Although ^15^ O-water is not commonly used for STS, Schwarzbach et al initially evaluated it with FDG and ^11^ C-aminoisobutyric acid ( ^11^ C-AIB) in 11 histologically proven STS patients, showing tracer uptake in tumors with partial overlap with blood vessels. 4 37 A subsequent study in 21 patients with primary and recurrent STS demonstrated higher tracer accumulation in tumors versus muscles and blood vessels, suggesting potential utility in detecting STS and recurrence. 38 However, its ultra-short physical half-life (122 seconds) and the requirement for an on-site cyclotron severely limit its feasibility for routine clinical application in STS. Lindholm et al evaluated ^15^ O-water and FDG for blood flow and glucose metabolism in nine patients with localized musculoskeletal tumors, concluding that tumor perfusion can be measured, though utility is variable. 39 Kenny et al assessed the correlation between ^18^ F-fluciclatide uptake and ^15^ O-water perfusion in patients with non-small cell lung cancer and melanoma, finding strong early correlation but no correlation at late time points, indicating that ^18^ F-fluciclatide retention is independent of perfusion. 40

### ^11^ C-Labeled Tracers

#### ^11^ C-Aminoisobutyric Acid

AIB is a synthetic, nonmetabolized amino acid that accumulates via the alanine-preferring transport system. 41 42 Conti et al evaluated AIB in patients with advanced malignant melanoma. 41 Schwarzbach et al also assessed AIB in STS, showing tumor accumulation with overlap with muscle and blood vessels. 4 37 38 Schmall et al demonstrated tumor detection in two patients with malignant fibrous histiocytoma using ^11^ C-AIB. 43

#### ^11^ C-Methionine

^11^ C-methionine ( ^11^ C-MET) accumulates in tumor cells through active transport and incorporation into protein fractions, lipids, and nucleic acids. 44 45 Later studies suggested uptake is primarily due to active transport rather than protein synthesis. 46 ^11^ C-MET demonstrates a higher tumor-to-background ratio (TBR) compared with FDG, particularly in tissues with low physiological protein synthesis, such as the brain and extremities, thereby improving lesion detectability and diagnostic confidence in these regions. Inoue et al compared ^11^ C-MET with FDG in malignant tumors, showing similar visualization but higher FDG uptake. 47 Zhang et al used ^11^ C-MET PET to monitor 15 chordoma patients before and after carbon-ion radiotherapy, showing reduced tumor uptake after treatment. 48 The tracer has also been used to predict survival in bone and STS post-carbon ion radiotherapy. 48 Ghigi et al found FDG superior to ^11^ C-MET in differentiating partial from complete response after neoadjuvant therapy. 49 Bredella et al reported that ^11^ C-MET improved detection of malignant peripheral nerve sheath tumors in neurofibromatosis type 1 patients, where FDG was equivocal. 50 PET imaging of non-small cell lung carcinoma also showed a correlation between ^11^ C-MET uptake and flow-cytometric parameters. 51

#### ^11^ C-Choline

Choline is essential for phospholipid biosynthesis and tumor proliferation. 52 Yanagawa et al and Zhang et al compared ^11^ C-choline with FDG, showing comparable sensitivity, shorter imaging time, and less bladder retention. 53 54 Tateishi et al demonstrated superior accuracy of ^11^ C-choline for preoperative staging and differentiation of benign versus malignant bone and soft tissue tumors. 55

#### ^11^ C-Tyrosine

L-tyrosine is an essential amino acid with high turnover in tumors, reflecting protein synthesis. 45 ^11^ C-tyrosine also provides superior tumor-to-background contrast compared with FDG in regions of low physiological protein synthesis, such as the brain and extremities, enhancing lesion detection and characterization. Kole et al studied ^11^ C-tyrosine in 55 patients with suspected malignant soft tissue lesions, finding better correlation with mitotic rate and proliferation than FDG, especially posttreatment. 56 van Ginkel et al demonstrated its utility in monitoring response after hyperthermic isolated limb perfusion in 17 patients, with posttreatment inflammation not affecting tumor detection. 57

### ^18^ F-Labeled Tracers

#### ^18^ F-Alpha-methyl-tyrosine

^18^ F-alpha-methyl-tyrosine (FMT) is an amino acid analogue accumulated via amino acid transport. 58 Watanabe et al and Ahmed et al demonstrated FMT's utility in differentiating benign from malignant musculoskeletal tumors, including schwannomas, and in preoperative planning. 59 60 61

#### ^18^ F-Fluorothymidine

^18^ F-fluorothymidine (FLT), a thymidine analogue, incorporates into DNA synthesis pathways, enabling assessment of tumor proliferation. 62 63 Cobben et al, Buck et al, Been et al, and Benz et al confirmed FLT's ability to visualize STS, grade tumors, and evaluate treatment response, including after hyperthermic isolated limb perfusion and neoadjuvant therapy. 63 64 65 66

#### ^18^ F-Fluoromisonidazole

^18^ F-fluoromisonidazole (FMISO) is a hypoxia-targeting tracer with retention determined by local oxygen tension. Koh et al first evaluated FMISO in human tumors. 67 Rajendran et al reported discordance between FMISO and FDG uptake in STS, indicating hypoxia and glucose metabolism are not always correlated. 68 69 Bentzen et al showed FMISO uptake did not accurately reflect tissue hypoxia measured by oxygen electrodes. 70 Muzi et al demonstrated the feasibility of image-derived FMISO activity as a surrogate for blood sampling in hypoxia quantification. 71

#### ^18^ F-Fluorodeoxyglucose

FDG reflects glucose metabolism via GLUT-1 transporters and hexokinase activity. 72 SUV-based quantitative metrics are highly sensitive to preanalytical and acquisition factors, particularly blood glucose levels and the timing of uptake/incubation, which can significantly influence measured tracer uptake. In addition, the lack of cross-platform harmonization between conventional PET/CT systems and newer digital or long axial field-of-view PET scanners remains a major challenge for standardized multicenter quantitative comparisons. FDG PET is widely used in STS for tumor detection, grading, biopsy guidance, prognostication, and early treatment response monitoring. 19 20 34 73 74 75 76 SUVmax, TLG, and volume-based PET markers provide independent prognostic information. 77 FDG PET is also sensitive for evaluating response to neoadjuvant chemotherapy and hyperthermic isolated limb perfusion.

## Summary

Multiple PET tracers—including ^15^ O-water, ^11^ C-AIB, ^11^ C-MET, ^11^ C-choline, ^11^ C-tyrosine, ^18^ F-FMT, ^18^ F-FLT, ^18^ F-FMISO, and ^18^ F-FDG—have been explored for STS evaluation. FDG PET remains the most commonly used clinical tracer, while others provide complementary information on tumor perfusion, amino acid transport, proliferation, and hypoxia, supporting diagnosis, treatment planning, monitoring, and prognostication.

Table 1 summarizes the main PET tracers used in STS, their targets, clinical applications, advantages, and limitations.

**Table 1:** PET tracer comparison in soft tissue sarcoma

PET tracer	Target/mechanism	Clinical applications	Advantages	Limitations
^15^ O-Water	Blood flow/perfusion	Assessment of tumor perfusion, recurrence detection, correlation with other tracers (FDG, ^18^ F-fluciclatide)	Quantitative measurement of tissue blood flow; early perfusion assessment	Not tumor-specific; short half-life (∼2 minutes); requires on-site cyclotron; overlap with blood vessels and muscles; limited STS studies
^11^ C-Aminoisobutyric acid (AIB)	Alanine-preferring amino acid transport	Detection of STS, tumor metabolism	Good tumor uptake; nonmetabolized tracer	Uptake overlaps with muscles and blood vessels; limited availability; short half-life (∼20 minutes)
^11^ C-Methionine (MET)	Amino acid transport, protein synthesis	Tumor detection, treatment monitoring, survival prediction	Sensitive to tumor metabolism; useful for treatment response (e.g., carbon-ion radiotherapy)	Short half-life (∼20 min); FDG often superior for therapy response; limited availability
^11^ C-Choline	Phospholipid synthesis, cell membrane proliferation	Tumor detection, preoperative planning, staging	Short imaging time; less bladder retention; differentiates benign versus malignant lesions	Limited availability; short half-life; less established than FDG for STS
^11^ C-Tyrosine (TYR)	Protein synthesis, amino acid transport	Assessing proliferation and mitotic rate; monitoring treatment response	Good correlation with mitotic activity; posttreatment inflammation minimally affects detection	Short half-life; limited clinical experience; overlaps with other tissues
^18^ F-Alpha-methyl-tyrosine (FMT)	Amino acid transport	Differentiating benign versus malignant tumors; musculoskeletal tumor assessment	Longer half-life than ^11^ C tracers (∼110 minutes); superior for benign versus malignant differentiation	Less widely available; grading of tumors may be less accurate than FDG
^18^ F-Fluorothymidine (FLT)	Cell proliferation/DNA synthesis	Tumor grading, treatment response monitoring	Evaluates proliferation; correlates with histopathology; can monitor response to chemotherapy or hyperthermic perfusion	Uptake may be lower than FDG; limited availability; less useful for initial staging
^18^ F-Fluoromisonidazole (FMISO)	Hypoxia	Detecting hypoxic tumor regions, therapy resistance assessment	Selective for hypoxic cells; perfusion-independent	Uptake may not reflect true hypoxia in STS; requires delayed imaging; less correlation with FDG; limited STS studies
^18^ F-Fluorodeoxyglucose (FDG)	Glucose metabolism	Tumor detection, grading, biopsy guidance, prognostication, therapy response	Widely available; high sensitivity for STS; correlates with tumor grade, mitotic activity, and necrosis	Cannot always differentiate low- versus high-grade STS; nonspecific uptake in inflammation; cannot fully predict histopathologic response

Abbreviation: STS, soft tissue sarcoma.

### Quantitative PET Metrics in Soft Tissue Sarcoma

Quantitative PET metrics have become indispensable in the assessment of tumor biology, therapy response, and prognosis in STS. Among these, the SUVmax, which represents the highest radiotracer concentration within a tumor, has been widely used to evaluate tumor aggressiveness, correlate with histopathological features such as mitotic activity and cellularity, and guide biopsy selection. 1 4 39 The mean standardized uptake value (SUVmean) provides a complementary measure of global tumor metabolism by averaging uptake over the entire lesion, thus reducing bias from focal areas of high tracer accumulation. 1 Beyond intensity metrics, MTV measures the volume of metabolically active tumor above a defined threshold, offering an objective assessment of tumor burden while excluding necrotic or hypometabolic regions. Bredella et al 78 and Cobben et al 63 demonstrated that a higher baseline MTV is associated with poorer outcomes, and reductions in MTV after therapy can serve as early indicators of treatment response. However, the clinical interpretation of SUVmax, MTV, and TLG is limited by a lack of standardization and inter-study variability, including differences in acquisition protocols, reconstruction parameters, segmentation methods, and threshold definitions, which may affect reproducibility and comparability across studies. 14 32 33

TLG, which integrates MTV and SUVmean, has emerged as a robust biomarker combining tumor burden and metabolic activity. High baseline TLG is correlated with higher recurrence risk and worse overall survival, while post-therapy reductions in TLG may predict therapeutic efficacy more sensitively than anatomical imaging alone. 30 38 Additionally, kinetic and dynamic PET parameters, obtained through time-resolved imaging and compartmental modeling, allow assessment of tracer delivery, perfusion, and metabolic flux within tumors. Lindholm et al 39 and Kenny et al 40 showed that dynamic PET distinguishes perfusion-limited from metabolism-limited uptake, providing mechanistic insights into tumor physiology and microenvironmental response to therapy.

The TBR further enhances lesion detection and characterization by normalizing tumor uptake to surrounding tissue activity, particularly useful in regions with physiologically high uptake, such as muscle or bone. Ioannidis and Lau 17 and Benz et al 16 reported that high TBR values are associated with aggressive tumor biology and improve detection sensitivity for heterogeneous STS. Collectively, these metrics—SUVmax, SUVmean, MTV, TLG, kinetic/dynamic parameters, and TBR—offer a comprehensive multidimensional assessment of STS. When applied in combination, they support biopsy guidance, treatment planning, early response evaluation, and prognostic stratification, highlighting the growing role of quantitative PET in personalized management of STS. 1 4 30 38 39 40 41 42

Table 2 provides definitions, clinical relevance, advantages, and limitations of the main PET metrics in STS.

**Table 2:** Quantitative PET metrics in soft tissue sarcoma

PET metric	Definition	Clinical relevance in STS	Advantages	Limitations
SUVmax	Maximum standardized uptake value; the highest radiotracer uptake in the tumor	Correlates with tumor aggressiveness, mitotic activity, histologic grade, and prognosis; guides biopsy and treatment planning	Simple to measure; widely validated; sensitive for detecting highly active tumor regions	Sensitive to noise and image reconstruction; reflects only a single voxel; may overestimate tumor activity in heterogeneous lesions
SUVmean	Average standardized uptake value across the entire tumor volume	Provides overall metabolic activity; complements SUVmax for therapy response assessment	Reduces bias from focal hotspots; more representative of global tumor metabolism	Requires accurate tumor segmentation; affected by partial volume effects
Metabolic tumor volume (MTV)	Volume of tumor tissue with uptake above a defined threshold	Quantifies metabolically active tumor burden; predicts prognosis and therapy response	Reflects tumor burden rather than focal activity; useful for treatment planning	Threshold selection can vary; may exclude hypometabolic but viable tumor areas
Total lesion glycolysis (TLG)	Product of MTV and SUVmean; represents combined tumor volume and metabolic activity	Predicts overall survival and treatment response; early biomarker of therapy efficacy	Integrates volume and metabolism; more robust than SUVmax alone	Dependent on accurate MTV delineation; affected by tumor heterogeneity and segmentation errors
Kinetic/dynamic parameters	Measures tracer uptake rate, perfusion, and metabolic flux over time using compartmental modeling	Differentiates perfusion-limited versus metabolism-limited uptake; provides mechanistic insights	Evaluates underlying tumor physiology; may improve early therapy response assessment	Requires dynamic PET acquisition and arterial input function; complex modeling; time-consuming
Tumor-to-background ratio (TBR)	Ratio of tumor uptake to background tissue uptake	Enhances detection in anatomically challenging regions; correlates with tumor aggressiveness	Normalizes uptake variability; improves lesion conspicuity	Dependent on accurate background selection; can vary with tissue physiology

Abbreviations: PET, positron emission tomography; STS, soft tissue sarcoma.

### Biological Heterogeneity and PET Phenotypes

STSs are a highly heterogeneous group of tumors, encompassing more than 70 distinct histologic subtypes, each with unique molecular signatures and clinical behaviors. 1 This biological heterogeneity is reflected in variable PET imaging phenotypes. FDG PET primarily measures glucose metabolism and is widely used for assessing tumor activity; however, it does not directly capture other key aspects of tumor biology, such as proliferation, amino acid metabolism, or hypoxia. 30 For example, discrepancies between FDG uptake and hypoxia-sensitive tracers such as FMISO have been reported, indicating that regions of high metabolic activity may not always correspond to hypoxic or treatment-resistant areas. 68

Multiparametric PET imaging, which combines FDG with proliferation markers (e.g., FLT) or microenvironment-targeted tracers (e.g., FMISO for hypoxia, ^15^ O-water for perfusion), allows a more comprehensive characterization of STS phenotypes. 39 Studies by Benz et al 79 and Buck et al 80 demonstrated that integrating metabolic and proliferation imaging provides complementary information that enhances tumor grading, treatment response assessment, and prognostication. Similarly, dynamic PET metrics capturing perfusion and tracer kinetics can identify regions of variable vascularization and metabolic heterogeneity within the same tumor, which may have implications for targeted therapy planning. 14

This multiparametric approach also addresses intratumoral heterogeneity, a key determinant of therapeutic resistance in STS. For instance, a tumor region may display high FDG uptake but low FLT retention, indicating metabolically active but poorly proliferating cells, whereas other regions may be hypoxic but metabolically quiescent. 79 81 Such insights can guide biopsy selection, delineate radiation therapy targets, and optimize neoadjuvant chemotherapy regimens. Furthermore, combining anatomical imaging with functional PET phenotypes enhances detection of aggressive subregions that might be overlooked by standard imaging modalities alone. 14

In summary, the integration of multiple PET tracers and quantitative imaging metrics provides a phenotypic map of STS biology, capturing variations in metabolism, proliferation, and microenvironmental factors. This multiparametric strategy not only improves diagnostic accuracy but also supports personalized treatment planning by identifying tumor subregions at high risk for progression or therapy resistance. 82 Future studies combining radiomics, PET heterogeneity metrics, and molecular profiling are likely to further refine PET-based phenotyping and predictive modeling in STS.

### Impact on Clinical Decision-Making

PET/CT imaging has increasingly influenced clinical management in STS by providing functional information that complements conventional anatomical imaging. One of the most significant applications is in biopsy guidance, where PET helps identify metabolically active regions within heterogeneous tumors, increasing the likelihood of obtaining representative tissue and accurate histopathologic diagnosis. 83 Beyond diagnosis, PET metrics allow for early prediction of treatment response, often preceding morphological changes detectable by size-based criteria such as RECIST. Studies by Stroobants et al 74 and Schuetze et al 75 demonstrated that reductions in FDG uptake after the first cycle of chemotherapy correlate with histopathologic response, offering clinicians an opportunity to adjust therapy in non-responding patients. Similarly, Benz et al 20 and Buck et al 84 reported that quantitative PET parameters, including SUVmax, TLG, and MTV, provide prognostic information that complements traditional histologic grading, particularly in high-grade or advanced STS, enabling improved risk stratification and personalized treatment planning.

In radiotherapy planning, PET/CT has been used to guide dose escalation to metabolically active tumor sub-volumes, thereby maximizing tumor control while sparing normal tissue. 85 The functional delineation of tumor margins is especially valuable in high-grade STS, where regions of aggressive biology may extend beyond the anatomic boundaries visualized on MRI or CT. Similarly, in surgical planning, PET can detect multifocal disease, skip lesions, or satellite nodules that are often occult on conventional imaging, allowing for more precise resection margins and potentially reducing local recurrence rates. 86

Recent advances in radiomics and AI have further expanded the clinical utility of PET imaging in STS. By extracting high-dimensional features from PET/CT images, radiomics can quantify intratumoral heterogeneity and correlate imaging phenotypes with molecular profiles, prognosis, and treatment response. 87 88 89 90 Machine learning models integrating PET radiomic signatures with clinical and histopathologic data have shown promise in predicting outcomes, potentially guiding individualized therapy strategies and improving patient selection for neoadjuvant or adjuvant treatments.

Overall, PET/CT has a multi-faceted impact on clinical decision-making in STS, from improving diagnostic accuracy and biopsy targeting to enhancing prognostication and treatment personalization. Its greatest value lies in high-grade or advanced tumors, where metabolic imaging can reveal biologically aggressive subregions that are critical for optimizing therapy, guiding radiotherapy and surgery, and potentially improving patient outcomes. 77 79 82 90 Continued integration of multiparametric PET imaging, quantitative metrics, and AI-driven analytics is likely to further refine clinical decision-making and enable truly personalized management strategies in STS.

## Limitations and Pitfalls

Despite the significant clinical utility of PET/CT in STS, several limitations must be acknowledged. The most widely used tracer, FDG, demonstrates nonspecific uptake, as inflammatory processes, post-surgical changes, or infection can mimic tumor activity, potentially leading to false-positive findings. 91 Additionally, partial volume effects in small lesions can result in underestimation of tracer uptake, limiting the sensitivity for detecting sub-centimeter or heterogeneous tumor regions. 92 Non-FDG PET tracers, including ^11^ C-amino acids, ^11^ C-choline, and FLT, offer enhanced specificity for proliferation, perfusion, or hypoxia; however, their limited availability, short half-lives, and high production costs restrict their routine clinical use to specialized centers. 93

Another challenge lies in the heterogeneity of sarcoma subtypes, which complicates the standardization of PET imaging protocols and quantitative metrics. Variability in tumor biology, grade, and metabolic activity across more than 70 histologic subtypes may lead to inconsistent SUV thresholds and cut-offs for response assessment. 79 Most published studies are limited by small patient cohorts, often including fewer than 20 to 50 subjects per study, reducing statistical power and generalizability of findings. 90

Moreover, the lack of standardized non-FDG response criteria hampers the interpretation of therapy response when using alternative tracers. While SUV-based metrics such as SUVmax, MTV, and TLG are widely applied, there is no universally accepted threshold for defining metabolic response across different tracers or sarcoma subtypes. 82 Finally, accessibility and cost considerations remain barriers to the widespread adoption of advanced PET tracers and multiparametric imaging, particularly in resource-limited settings, limiting their integration into routine clinical workflows. 94 The ready electronic availability of the patients' medical records may alleviate some of the potential risks of an incomplete medical history as submitted with the PET/CT request.

In conclusion, while PET imaging offers valuable functional insights into tumor biology, clinicians must remain aware of its technical, biological, and logistical limitations. Recognition of these pitfalls is essential for accurate interpretation, appropriate tracer selection, and integration of PET data with conventional imaging and histopathology to optimize patient management in STS.

## Emerging Radiotracers and Future Directions

The development of novel PET radiotracers and multimodal imaging approaches represents a promising frontier in personalized STS management. Beyond FDG, emerging tracers, such as FLT, fibroblast activation protein inhibitor (FAPI), and FMISO, enable more specific evaluation of proliferation, tumor stroma, and hypoxia, respectively, providing complementary insights into tumor biology. 95 96 Integrating multiple tracers in a multitracer PET protocol allows simultaneous assessment of glucose metabolism, cellular proliferation, and the tumor microenvironment, which may improve therapy planning, detect resistant sub-volumes, and facilitate early response assessment.

The combination of PET with MRI offers further advantages by adding superior soft tissue contrast, functional sequences such as diffusion-weighted imaging, and dynamic contrast-enhanced imaging, improving localization and characterization of heterogeneous lesions. However, despite encouraging results, PET/MRI remains an evolving technique and its role in routine STS management has not yet been fully established, requiring further prospective validation. 8 97 Additionally, radiomics and AI-driven analysis can extract high-dimensional imaging features, enabling predictive modeling of histologic grade, response to therapy, and patient outcomes, potentially surpassing conventional SUV metrics in prognostic accuracy. While these approaches have demonstrated considerable promise in preliminary studies, they remain largely investigational because of limited prospective validation, methodological variability, and lack of standardized implementation frameworks. 31 98

Emerging clinical evidence from early-phase studies supports the potential utility of FAPI PET/CT in sarcoma imaging, with initial diagnostic and theranostic applications reported in recent studies. 99 100 101 102 103 104 However, the current evidence base remains limited and primarily derived from small cohorts and early clinical experience. FAPI PET/CT has shown promising tumor-to-background contrast and improved lesion conspicuity in selected stromal-rich sarcoma subtypes, suggesting potential advantages in tumor delineation compared with FDG PET/CT; however, these findings require validation in larger prospective comparative studies. 101 First-in-human and early dose-escalation studies of ^177^ Lu-FAPI radioligand therapy indicate the feasibility of therapeutic targeting of FAP-expressing tumors, providing proof-of-concept for a theranostic approach in sarcoma, although clinical efficacy and long-term outcomes remain under investigation.

At a biological level, FAPI PET/CT has recently emerged as a promising imaging modality in STS, targeting cancer-associated fibroblasts within the tumor microenvironment. Early evidence suggests that FAPI PET/CT provides superior tumor delineation compared with FDG in several sarcoma subtypes, particularly in tumors with low-to-moderate glucose metabolism and high stromal content. In addition, its high tumor-to-background contrast may improve lesion detectability and facilitate more accurate tumor mapping for surgical and radiotherapy planning. Recent systematic evidence further supports its diagnostic and theranostic potential in STS, highlighting its role as a complementary imaging biomarker to FDG PET/CT. Beyond its diagnostic role, FAPI also demonstrates growing theranostic potential, enabling both imaging and potential targeted radionuclide therapy in FAP-expressing tumors. Emerging evidence suggests that uptake varies across histological subtypes, with particularly intense FAPI expression reported in solitary fibrous tumors, as well as other stromal-rich sarcomas, reflecting their high fibroblast content. These findings highlight its value not only for tumor delineation but also for patient selection in future FAPI-based targeted therapeutic strategies. Recent systematic evidence further supports these applications and underscores its complementary role to FDG PET/CT in STS management.

In summary, the future of STS imaging lies in multiparametric, personalized approaches, leveraging novel radiotracers, hybrid imaging modalities, quantitative radiomics, and molecular insights to refine diagnosis, optimize therapy guidance, and improve prognostication. While promising, these strategies require validation in larger multicenter studies to establish standardized protocols, cost-effectiveness, and clinical impact before routine implementation in sarcoma management.

## Conclusion

PET/CT is an essential imaging modality in STS, providing functional and metabolic information that complements anatomical imaging in staging, biopsy guidance, treatment response assessment, and prognostication. ^18^ F-FDG PET/CT remains the established clinical standard. Emerging radiotracers, multiparametric imaging, and advanced analytical approaches such as radiomics and AI expand biological characterization and improve assessment of tumor heterogeneity. However, these techniques remain investigational and require further prospective validation and standardization before routine clinical adoption in STS.
